# LOX-1 Plays an Important Role in Ischemia-Induced Angiogenesis of Limbs

**DOI:** 10.1371/journal.pone.0114542

**Published:** 2014-12-16

**Authors:** Takeru Shiraki, Takuma Aoyama, Chiharu Yokoyama, Yuka Hayakawa, Toshiki Tanaka, Kazuhiko Nishigaki, Tatsuya Sawamura, Shinya Minatoguchi

**Affiliations:** 1 Department of Cardiology, Gifu University Graduate School of Medicine, Gifu, Japan; 2 Department of Vascular Physiology, National Cerebral and Cardiovascular Center Research Institute, Suita, Japan; 3 Department of Physiology, Shinshu University School of Medicine, Matsumoto, Japan; University of Illinois at Chicago, United States of America

## Abstract

LOX-1, lectin-like oxidized low-density lipoprotein (LDL) receptor-1, is a single transmembrane receptor mainly expressed on endothelial cells. LOX-1 mediates the uptake of oxidized LDL, an early step in atherosclerosis; however, little is known about whether LOX-1 is involved in angiogenesis during tissue ischemia. Therefore, we examined the role of LOX-1 in ischemia-induced angiogenesis in the hindlimbs of LOX-1 knockout (KO) mice. Angiogenesis was evaluated in a surgically induced hindlimb ischemia model using laser Doppler blood flowmetry (LDBF) and histological capillary density (CD) and arteriole density (AD). After right hindlimb ischemia, the ischemic/nonischemic hindlimb blood flow ratio was persistently lower in LOX-1 KO mice than in wild-type (WT) mice. CD and AD were significantly smaller in LOX-1 KO mice than in WT mice on postoperative day 14. Immunohistochemical analysis revealed that the number of macrophages infiltrating ischemic tissues was significantly smaller in LOX-1 KO mice than in WT mice. The number of infiltrated macrophages expressing VEGF was also significantly smaller in LOX-1 KO mice than in WT mice. Western blot analysis and ROS production assay revealed that LOX- KO mice show significant decrease in Nox2 expression, ROS production and HIF-1α expression, the phosphorylation of p38 MAPK and NF-κB p65 subunit as well as expression of redox-sensitive vascular cell adhesion molecule-1 (VCAM-1) and LOX-1 itself in ischemic muscles, which is supposed to be required for macrophage infiltration expressing angiogenic factor VEGF. Reduction of VEGF expression successively suppressed the phosphorylation of Akt and eNOS, which accelerated angiogenesis, in the ischemic leg of LOX-1 KO mice. Our findings indicate that LOX-1 plays an important role in ischemia-induced angiogenesis by 1) Nox2-ROS-NF-κB activation, 2) upregulated expression of adhesion molecules: VCAM-1 and LOX-1 and 3) promoting macrophage infiltration, which expresses angiogenic factor VEGF.

## Introduction

Angiogenesis is modulated by a variety of factors: angiogenic growth factors, cytokines, inflammatory leukocytes, bone marrow-derived progenitor cells, extracellular matrices, vasoactive substances and NADPH oxidase [1]–[5]. In addition, it has been reported that macrophage infiltration early after ischemia is an important trigger for promoting ischemia-induced angiogenesis, since inflammatory cells release the angiogenic growth factor vascular endothelial growth factor (VEGF) [6], [7].

LOX-1 is a type II integral membrane glycoprotein with a short N-terminal cytoplasmic domain, a single transmembrane domain, a short ‘neck’ or stalk region and an extracellular C-type lectin-like fold [8], [9]. LOX-1 was first identified as an endothelial-specific scavenger receptor but was also detected on macrophages, smooth muscle cells, monocytes and platelets later [8]–[15].

In early atherosclerotic lesions, LOX-1 levels are elevated both within the intima and in the endothelium surrounding the lesion, suggesting that LOX-1 is involved in endothelial dysfunction and the initiation and growth of atherosclerotic plaques [16]–[19]. On the other hand, it has been reported that: (1) oxidized LDL, which is a ligand of LOX-1, markedly increased the expression of VEGF mRNA and increased the release of VEGF protein and its effects which were significantly suppressed by anti-LOX-1 antibody pretreatment, (2) LOX-1 is an adhesion molecule involved in leukocyte recruitment, (3) the expression of VCAM-1 and ICAM-1, as well as the number of macrophages around blood vessels, were significantly increased in LOX-1 TG/ApoE KO mice compared with control mice [17], [20], [21]. These reports suggest that activated LOX-1 has various aspects in cardiovascular diseases.

Interestingly, previous studies have shown that LOX-1 is involved in the production of oxidant stress and inflammation after ischemia of the heart, suggesting that LOX-1 can be activated in ischemic tissues [22], [23]. Considering that inflammation is vital for ischemia-induced angiogenesis, LOX-1 may play an important role in angiogenesis after ischemia; however, little is known as to whether LOX-1 plays a role in the process of ischemia-induced angiogenesis. Accordingly, taking advantage of genetically modified LOX-1 KO mice, we examined in the present study whether LOX-1 plays a role in promoting ischemia-induced angiogenesis.

## Results

### Laser Doppler blood flow analysis

**Figure 1:**
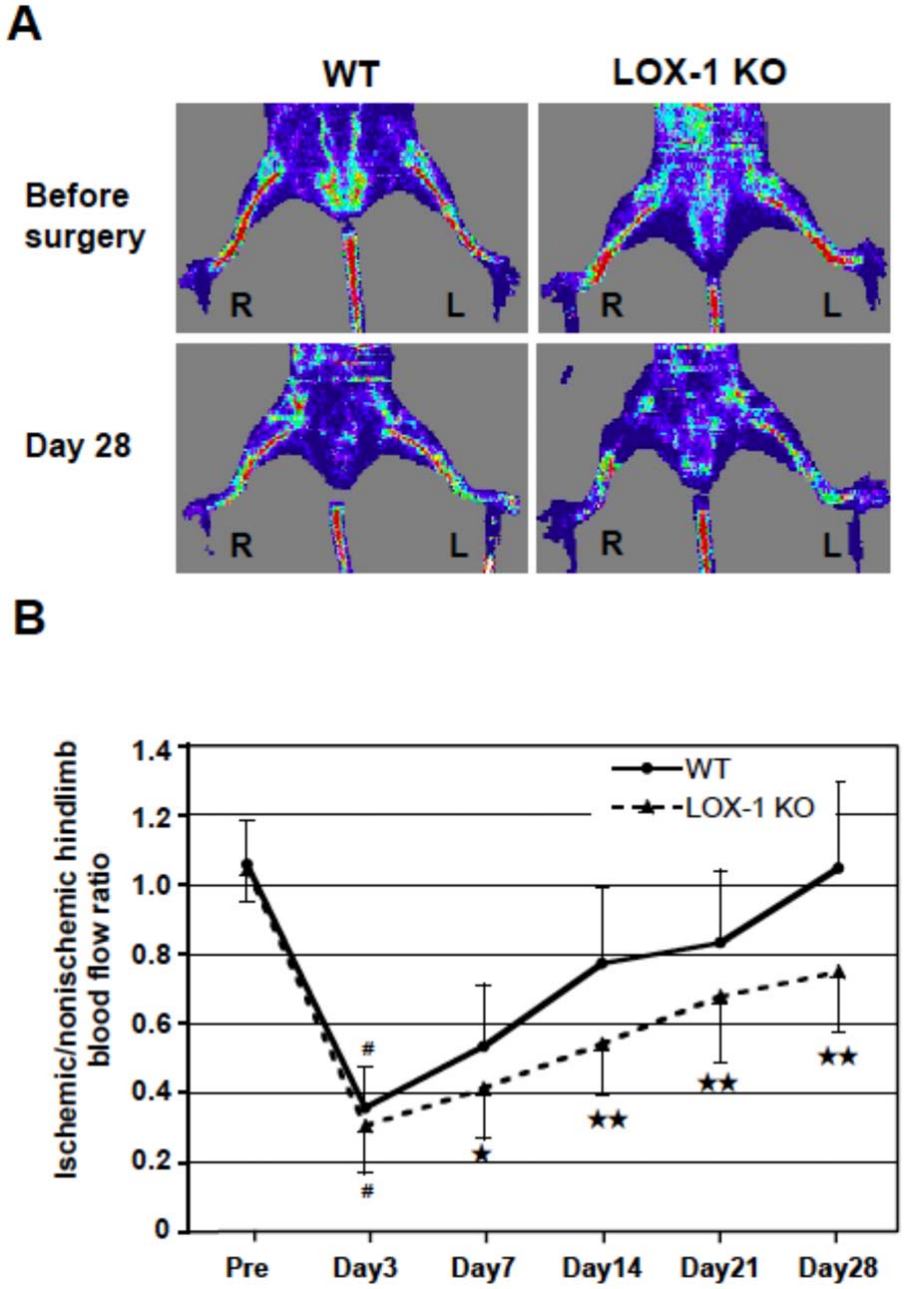
Laser Doppler blood flow analysis of WT and LOX-1 KO mice. A: Representative images of LDBF just before and day 28 after surgery revealed hindlimb blood flow of the ischemic (R: right)/nonischemic (L: left) conditions in a supine position. B: Ischemic/nonischemic hindlimb blood flow ratio (mean±SD) were measured before (Pre), and on days 3, 7, 14, 21 and 28 after right femoral artery ligation. Results are expressed as the ratio of the ischemic hindlimb to nonischemic limb perfusion. ^★^, P<0.05 vs. WT; ^★★^, P<0.01 vs. WT; ^#^, P<0.01 vs. Pre (before surgery), n = 25.

For evaluation of blood flow recovery after ligation of the femoral artery of WT and LOX-1 KO mice, LDBF analysis was performed on the preoperative state and on days 3, 7, 14, 21 and 28. Representative images of blood flow as measured by LDBF are shown in Fig. 1A. The upper part of Fig. 1A shows the state of hindlimb blood flow before surgery. No difference was observed between the right and left hindlimb blood flow in either the WT or the LOX-1 KO mice. The lower part of Fig. 1A shows the state of blood flow in the hindlimbs of mice at 28 days after surgery. Blood flow in the ischemic (R: right) hindlimb of WT mice recovered to almost the same level as the blood flow in the nonischemic (L: left) hindlimb at 28 days after induction of hindlimb ischemia. However, blood flow in the ischemic hindlimb (R) was markedly reduced in LOX-1 KO mice compared with the level in the nonischemic hindlimb (L) on the same day.

Fig. 1B and Table 1 summarize the calculated ischemic/nonischemic hindlimb blood flow ratio. After the surgical induction of right hindlimb ischemia, the blood flow ratio significantly decreased in both the WT mouse and the LOX-1 KO mouse on postoperative day 3 compared with the flow before surgery (pre-surgery vs. day 3: WT p<0.01; LOX-1 KO p<0.01), showing no significant differences in the ratio between the two groups on postoperative day 3 (WT vs. LOX-1 KO, n.s.). Thus, the severity of induced hindlimb ischemia was comparable between the two groups. However, the ischemic/nonischemic hindlimb blood flow ratios on postoperative days 7, 14, 21 and 28 were significantly smaller in LOX-1 KO mice than in WT mice (WT vs. LOX-1 KO: day 7, p<0.05; day 14, p<0.01; day 21, p<0.01; and day 28, p<0.01).

**Table 1:** Ischemic/nonischemic hindlimb blood flow ratio (mean±SD) measured by laser Doppler blood flow analysis.

	Pre	Day 3	Day 7	Day 14	Day 21	Day 28
**WT (N = 25)**	1.06±0.13	0.36±0.12	0.53±0.18	0.77±0.22	0.83±0.21	1.05±0.25
**LOX-1 KO (N = 25)**	1.00±0.09	0.31±0.14	0.41±0.14	0.54±0.15	0.68±0.19	0.75±0.17
**P-value**	0.74	0.17	0.01	<0.001	0.007	<0.001

For evaluation of blood flow recovery after ligation of the femoral artery of WT and LOX-1 KO mice, LDBF analysis was performed on the preoperative state and on days 3, 7, 14, 21 and 28. Data are expressed as the mean ± SD. n = 25.

### Tissue capillary and arteriole density

**Figure 2:**
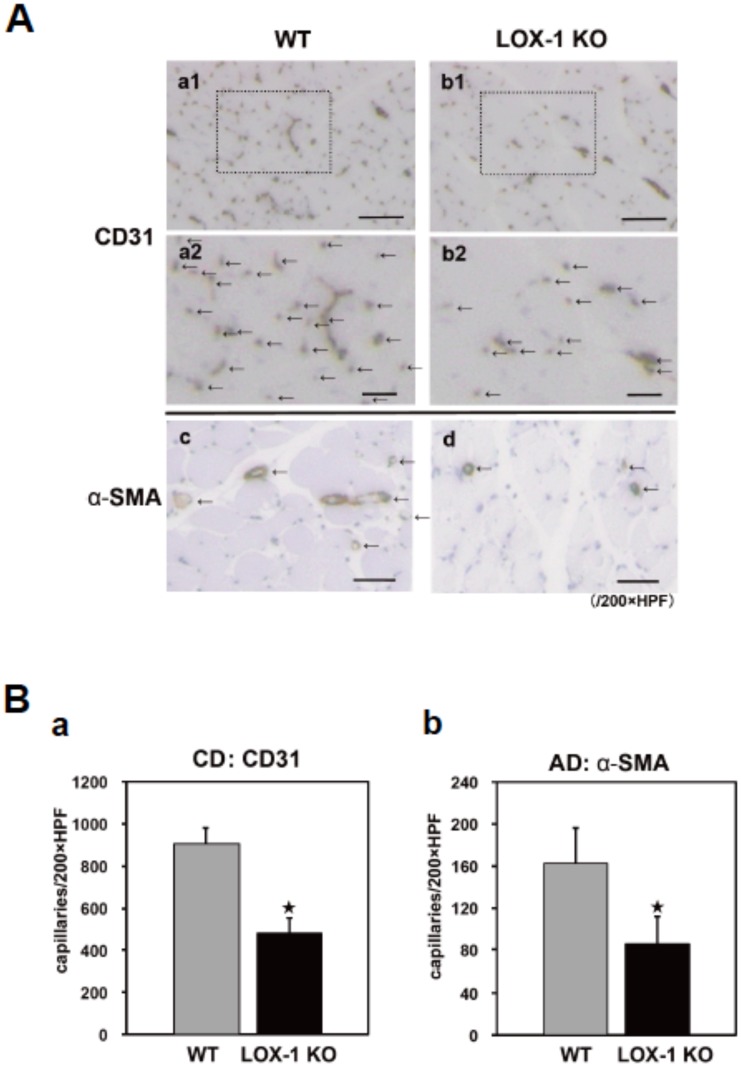
Determination of capillary density (CD) and arteriole density (AD). A: Representative photographs to show CD and AD evaluated by histological examination of 20 randomly selected fields of tissue sections. CD in the ischemic tissues was immunostained with CD31 (an endothelial cell marker; brown, a1,2 and b1,2 and arrows, a2 and b2) and AD was anti-α-smooth muscle actin (α–SMA: an arteriole marker; brown and arrows, c and d) in the ischemic tissues of the gastrocnemius muscles on postoperative day 14 in high-power microscopic fields (x200). The number of vessels was reduced in LOX-1 KO mice (b1,2) compared with that in WT mice (a1,2) in CD31 staining (arrows in a2 and b2: the area of a1 and b1 surrounded by dotted line) and the number of arterioles stained with α–SMA (arrows in c and d) was also reduced in LOX-1 KO mice (d) compared with that in WT mice (c), Original bars; a1, b1: 300 µm a2, b2: 100 µm, c, d: 300 µm. B: CD and AD (mean±SD) were quantitatively assessed by histological examination of 20 randomly selected fields of tissue sections stained with CD31 staining (a) and α–SMA staining (b). The calculated capillary and arteriole density (capillaries per x200 HPF, arterioles per x200 HPF) were significantly lower in LOX-1 KO mice than in WT mice. ^★^, P<0.01 vs. WT, n = 20.

To evaluate LOX-1-mediated angiogenesis in the ischemic hindlimb, the tissue capillary and arterole density were measured using immunohistochemistry. Representative photomicrographs of histological sections stained with anti-CD31 Ab (an endothelial cell marker; brown in Fig. 2A a1, 2 and b1, 2 and arrows in Fig. 2A a2 and b2) and anti-α-SMA Ab (an arteriole marker; brown and arrows in Fig. 2A c and d) are shown in Fig. 2A. CD31-positive capillaries and α-SMA-positive arterioles were identified in sections of the gastrocnemius muscle tissues on postoperative day 14. The number of vessels in slides stained with CD31 or α-SMA Ab in one microscopic field, which represents capillary density (CD) or arteriole density (AD), was reduced significantly in LOX-1 KO mice (Fig. 2A b1, 2 and d) compared with that in WT mice (Fig. 2A a1, 2 and c). Fig. 2B shows quantitative results for the number of vessels stained with CD31 or α-SMA Ab (/200x field) and reveals that CD and AD were significantly lower in LOX-1 KO mice than in WT mice (CD31: WT vs. LOX-1 KO: 906.6±71.0 vs. 481.1±72.6, p<0.01, α-SMA: WT vs. LOX-1 KO: 163.6±33.2 vs. 85.0±27.7, p<0.01). There was no significant difference between the two groups in CD and AD in the nonischemic hindlimb skeletal muscles (data not shown).

### Effect of ischemia on expression of LOX-1 and effect of deletion of LOX-1 on expression of VEGF, VEGFR2, HIF-1α, Nox2 and generation of ROS

**Figure 3:**
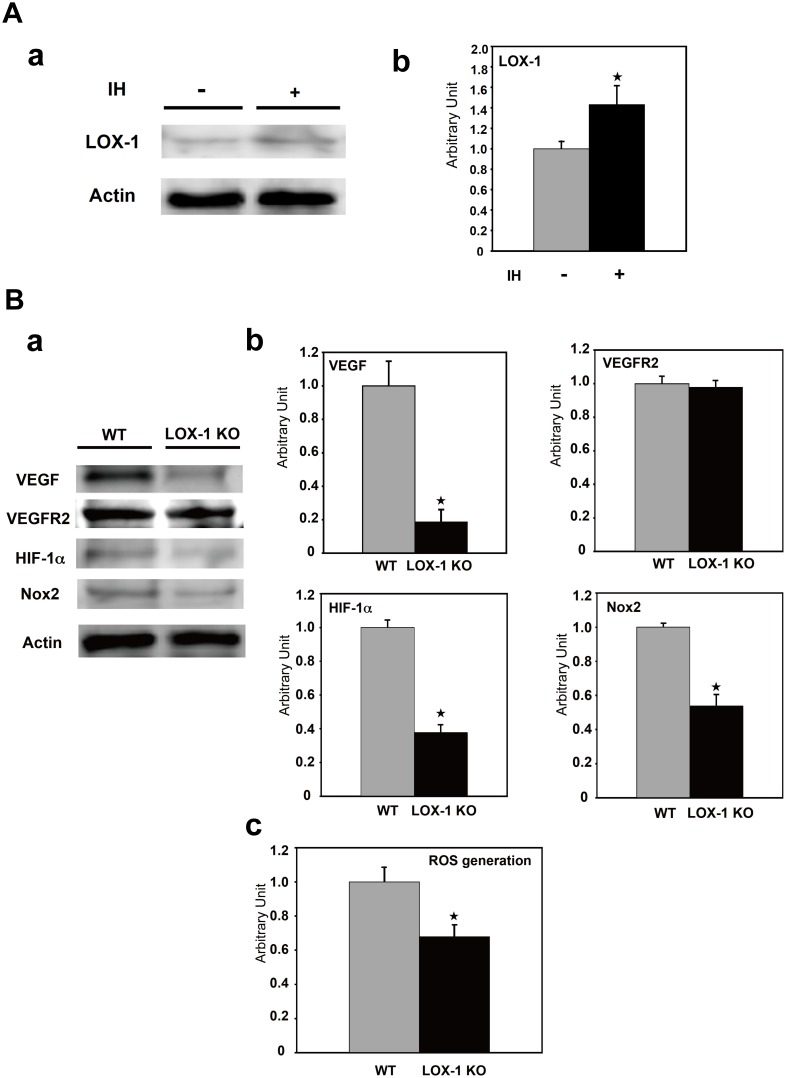
Expression of LOX-1, VEGF VEGFR2, HIF-1α, Nox2 and generation of ROS. **A. Effect of ischemia on expression of LOX-1.** a: The expression of LOX-1 was analyzed in the ischemic/nonischemic hindlimb of WT mice by Western blotting. Upregulation of LOX-1 expression in the ischemic hindlimb tissue on postoperative day 7 after ligation compared with in the nonischemic hindlimb of WT mice. Actin was used as loading control. IH: ischemia. b: Densitometric measurements (mean±SD) were performed to evaluate the fold increase in LOX-1 protein. The measured value is expressed as a value relative to the protein level of WT mice. IH: ischemia, ^★^, P<0.05 vs. WT, n = 4. **B. Effect of deletion of LOX-1 on expression of VEGF, VEGFR2, HIF-1α, Nox2 and generation of ROS.** a: The expression of VEGF, VEGFR2, HIF-1α and Nox2 in the ischemic hindlimb was analyzed by Western blotting. Suppression of VEGF, HIF-1α and Nox2 expression in the ischemic hindlimb tissue of LOX-1 KO mice on postoperative day 7 after ligation compared with that in WT mice. Actin was used as loading control. b: Densitometric measurements (mean±SD) were performed to evaluate the fold increase in VEGF, VEGFR2, HIF-1α and Nox2 protein. The measured value is expressed as a value relative to the protein level of WT mice. ^★^, P<0.01 vs. WT, n = 4. c: ROS generation was measured in the ischemic hindlimb of WT mice and LOX-1 KO mice. ^★^, P<0.01 vs. WT, n = 4.

In order to examine whether LOX-1 is upregulated in the ischemic hindlimb of WT mice, we examined the expression of LOX-1 protein by Western blot analysis. Western blot analysis showed that LOX-1 protein expression in the ischemic hindlimb was significantly increased compared with that in the nonischemic hindlimb in WT mice (Fig. 3A a) on postoperative day 7. As shown in Fig. 3A b, LOX-1 protein content in the ischemic hindlimb was increased to about 43% of that in the the nonischemic hindlimb in WT mice (ischemic vs. nonischemic, P<0.05).

Next, to confirm the effect of deletion of LOX-1 on the expression of angiogenic factor, we examined the expression of VEGF protein: the most powerful angiogenic factor. Western blot analysis showed that VEGF protein expression in hindlimb tissues after ischemia was significantly suppressed in LOX-1 KO mice compared with that in WT mice (Fig. 3B a) on postoperative day 7. As shown in Fig. 3B b, VEGF protein content in LOX-1 KO mice was reduced to about 19% of that in WT mice (WT vs. LOX-1 KO, P<0.01). Then we evaluated VEGF receptor-2 (VEGFR2) protein levels in the ischemic tissues of both mice by Western blot analysis. There were no significant differences of VEGFR2 protein levels between WT mice and LOX-1 KO mice (Fig. 3B a and b).

In order to address the physiological role of LOX-1 activation in the ischemic hindlimb, we examined the expressions of HIF-1α, an important target of hypoxia, NADPH oxidase 2 (Nox2: a major sources of ROS) and reactive oxygen species (ROS) generation. Nox2 expression was significantly suppressed in LOX-1 KO mice compared with WT mice (WT vs. LOX-1 KO, P<0.01, Fig. 3B a and b). Consistent with reduced Nox2 expression in LOX-1 KO mice, ROS generation in the ischemic hindlimb of LOX-1 KO mice was significantly lower than that of WT mice (WT vs. LOX-1 KO, P<0.01, Fig. 3B c).

HIF-1α expression levels were also lower in LOX-1 KO mice than in WT mice as well as Nox2 expression level as shown in Fig. 3B a and b (WT vs. LOX-1 KO, P<0.01).

### Effect of deletion of LOX-1 on signaling pathways (p38 MAPK, NF-κB p65 subunit, Akt, eNOS, ERK and SAPK) and NF-κB dependent inducible gene

We examined the effect of deletion of LOX-1 on its downstream signaling pathways after ischemic treatment. At first, we assessed both total and phosphorylated p38 MAPK expression and p65 subunit of NF-κB expression by Western blot analysis.

**Figure 4:**
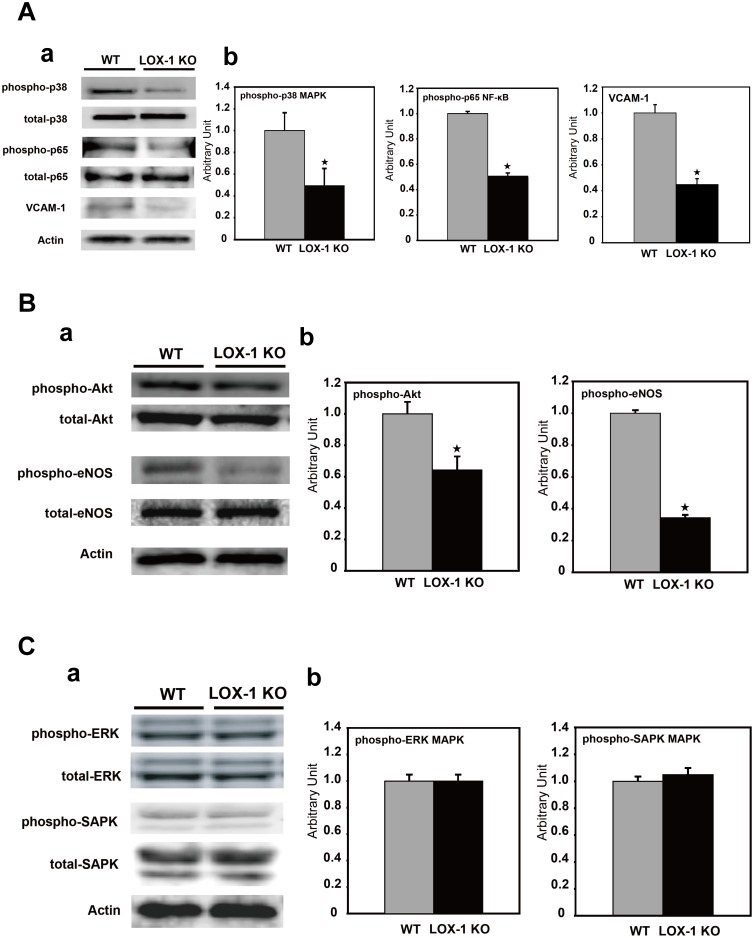
Effect of deletion of LOX-1 on signaling pathways and NF-κB dependent gene. **A: Effect of deletion of LOX-1 on signaling pathways (p38 MAPK and NF-κB p65 subunit) and VCAM-1: NF-κB dependent adhesion molecule.** a: The expression of total and phosphorylated forms of p38 MAPK and NF-κB p65 subunit and VCAM-1 in the ischemic hindlimb was analyzed by Western blotting. Phosphorylation of p38 MAPK and NF-κB p65 subunit was suppressed in the ischemic hindlimb tissue of LOX-1 KO mice compared with that in WT mice as well as VCAM-1 protein, but their total proteins were equivalent between WT mice and LOX-1 KO mice. Actin was used as loading control. b: Densitometric measurements (mean±SD) were performed to evaluate the fold increase in phosphorylation of p38 MAPK and NF-κB p65 subunit and VCAM-1. The measured value is expressed as the value relative to the protein level of WT mice. Actin was used as loading control. ^★^, P<0.01 vs. WT, n = 4. **B: Effect of deletion of LOX-1 on signaling pathways (Akt and eNOS).** a: The expression of total and phosphorylated forms of Akt and eNOS in the ischemic hindlimb was analyzed by Western blotting. Phosphorylation of Akt and eNOS was suppressed in LOX-1 KO mice compared with that in WT mice, but their total proteins were equivalent between WT mice and LOX-1 KO mice. Actin was used as loading control. b: Densitometric measurements (mean±SD) were performed to evaluate the fold increase in phosphorylation of Akt and eNOS. Both phospho-proteins were significantly suppressed in LOX-1KO mice compared with those in WT mice. The measured value is expressed as a value relative to the protein level of WT mice. ^★^, P<0.01 vs. WT, n = 4. **C: Effect of deletion of LOX-1 on signaling pathways (ERK and SAPK).** a: The expression of total and phosphorylated forms of ERK and SAPK in the ischemic hindlimb was analyzed by Western blotting. No differences were observed in phospho-ERK and phospho-SAPK protein expression levels between KO mice and WT mice, as well as both total proteins. Actin was used as loading control. b: Densitometric measurements (mean±SD) were performed to evaluate the fold increase in phosphorylation of ERK and SAPK. Phospho-ERK and phospho-SAPK proteins were quantitatively equivalent. The measured value is expressed as a relative value relative to the protein level of WT mice. ^★^, P<0.01 vs. WT, n = 4.

As seen in the upper part of Fig. 4A a, Western blot analysis showed that the expression of phosphorylated p38 MAPK, which is a signal downstream of LOX-1, was suppressed in ischemic tissue of LOX-1 KO mice compared with that in WT mice. When we quantified the amount of protein, phosphorylated p38 MAPK in LOX-1 KO mice was inhibited to 49% of that in WT mice (WT vs. LOX-1 KO, P<0.01, Fig. 4A a, b).

The phosphorylated p65 subunit of NF-κB in the ischemic tissues of LOX-1 KO mice was significantly suppressed by 51% compared with that in WT mice, as well as phosphorylated p38 MAPK (WT vs. LOX-1 KO, P<0.01, Fig. 4A a, b).

Next we examined the expression level of VCAM-1: NF-κB dependent inducible gene in the ischemic hindlimb in two kinds of mice. As shown in Fig. 4A a and b, VCAM-1 expression in the ischemic hindlimb of LOX-1 KO mice was significantly suppressed by 45% compared with that of WT mice (WT vs. LOX-1 KO, P<0.01).

Next, we evaluated the expression of both total and phosphorylated Akt and eNOS protein, which are related to angiogenesis as downstream signaling molecules of VEGF.

The total amounts of Akt and eNOS were similar between WT mice and LOX-1 KO mice. However, Akt and eNOS phosphorylation was significantly suppressed in LOX-1 KO mice compared with that in WT mice (Fig. 4B a). Quantification analysis showed that Akt phosphorylation in LOX-1 KO mice was reduced to 64% of that in WT mice. Furthermore, phosphorylation of eNOS in LOX-1 KO mice was suppressed to 37% of that in the WT mice (WT vs. LOX-1 KO, P<0.01, Fig. 4B b).

In addition to p38 MAPK, we examined the other two MAPKs, ERK and SAPK.

There were no significant differences in phosphorylated ERK and SAPK proteins between WT mice and LOX-1 KO mice, as well as none in their total proteins (Fig. 4C a, b).

### Immunohistochemical localization of VEGF and macrophages

**Figure 5:**
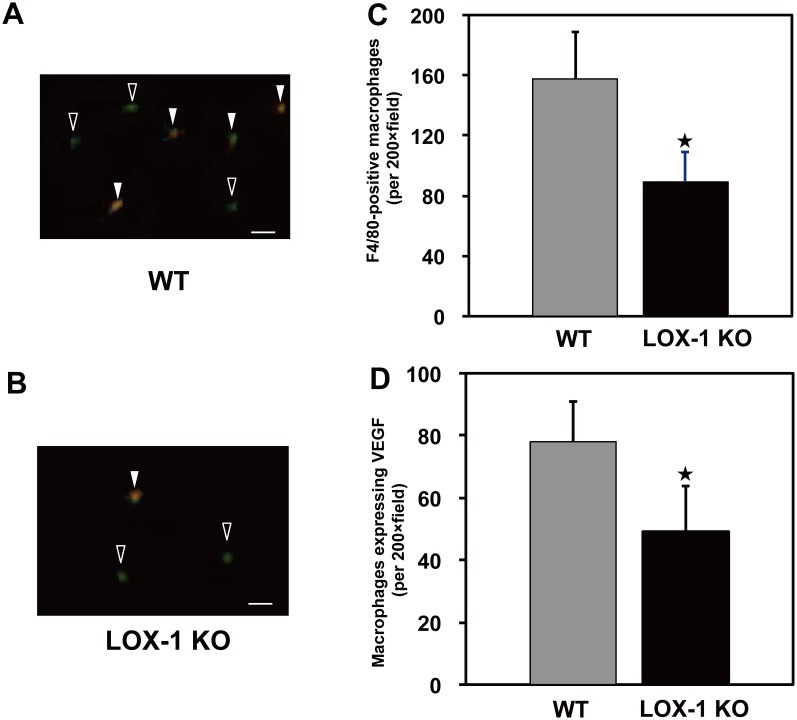
Two-color immunofluorescence staining. Infiltrated macrophages (green) and VEGF protein (orange) in ischemic gastrocnemius muscle sections on postoperative day 3 after surgery. In the merged pictures (A and B), there were some overlaps between macrophage infiltration and VEGF expression, suggesting that these infiltrated macrophages produce and release VEGF protein. Quantitative data indicated both macrophage infiltration (C) and macrophages expressing VEGF (D) and both of these were significantly less in LOX-1 KO mice than in WT mice. Open arrow: macrophage without VEGF expression (green), Closed arrow: macrophage (green) with VEGF expression (orange), Original bars  = 50 µm. ^★^, P<0.01 vs. WT, n = 20.

In order to identify which cells secrete VEGF, we carried out double-immunostaining analysis for VEGF and macrophages using antiserum. Representative photographs of double immunostaining of mouse leg tissues are shown in Fig. 5A (WT) and Fig. 5B (LOX-1 KO). Macrophages that were stained green by F4/80 monoclonal antibody were frequently observed in ischemic tissue of WT mice, but infiltration of macrophages was suppressed in LOX-1 KO mice compared with that in WT mice.

On the other hand, infiltrated F4/80-positive macrophages (green) were co-stained with anti-VEGF mAb (orange), indicating that infiltrated macrophages produce VEGF. Fig. 5A and 5B show that the number of macrophages producing VEGF (closed arrows) was less in LOX-1 KO mice than in WT mice. Quantitative analysis showed first that the number of infiltrated F4/80-positive macrophages was significantly smaller in LOX-1 KO mice than in WT mice, as shown in Fig. 5C (WT vs. LOX-1 KO: 157.9±30.7 vs. 89.3±20.4, p<0.01), and second, that the number of VEGF-positive macrophages was also smaller in LOX-1 KO mice than in WT mice (WT vs. LOX-1 KO: 77.7±13.4 vs. 49.6±15.7, p<0.01), as shown in Fig. 5D.

## Discussion

LOX-1 was originally identified as the major endothelial scavenger receptor for oxidized LDL, but was subsequently detected on other cell types such as smooth muscle cells and macrophages [10], [14]. LOX-1 has been increasingly linked to atherosclerotic plaque formation [18] and transgenic mouse models of gene knockout or LOX-1 overexpression also suggest that LOX-1 contributes to atherosclerotic plaque formation [24], [25].

On the other hand, LOX-1 ablation reduced myocardial infarct size and improved cardiac function after ischemia-reperfusion [22], [23]. It suggests that LOX-1 may function not only as an oxidized LDL receptor but also as a modulator of ischemic heart tissues. However, there are few reports demonstrating the role of LOX-1 as an angiogenic molecule in other ischemic tissues. Therefore, we investigated whether LOX-1 modulates angiogenesis in ischemic tissue using an ischemic hindlimb model of WT mice and LOX-1 KO mice.

Interestingly, we found upregulated LOX-1 expression in the ischemic hindlimb, suggesting that ischemic status must enhance the physiological function of LOX-1 (Fig. 3A a, b). Importantly, we found that blood flow recovery in the hindlimb after ligation of femoral artery was significantly suppressed in LOX-1 KO mice compared with that in WT mice (Fig. 1A, B). This suggests that LOX-1 is involved in blood flow recovery in an ischemic hindlimb. Recently other groups have reported that enhanced healing/regeneration after ischemia are due to increased densities of capillaries and arterioles in the ischemic hindlimb [26]–[29]. Therefore, we examined it and found that CD (staining with anti-CD31: capillary) and AD (staining with anti-α-SMA Ab: arteriole) in the ischemic hindlimb of LOX-1 KO mice were significantly decreased compared with that in WT mouse hindlimbs (Fig. 2A, B). This indicates that deletion of LOX-1 inhibits blood flow recovery via deceleration of arteriogenesis and angiogenesis after hindlimb ischemia.

LOX-1 activation rapidly elevates ROS level such as superoxide anions and hydrogen peroxide via activation of a membrane-bound NADPH oxidase [22], [30]–[33]. Recently it has been reported that Nox2-derived ROS are involved in postischemic mobilization of bone marrow cells (stem and progenitor cells) and revascularization [3]. We found the suppression of Nox2 expression, ROS generation and HIF-1α expression in the ischemic hindlimb of LOX-1 KO mice in this experiment (Fig. 3B a, b, c). Therefore, deletion of LOX-1 is supposed to reduce ROS-HIF-1α-VEGF pathway for neovascularization. Simultaneously Nox2-dependent ROS generation by activated LOX-1 stimulates intracellular signaling, namely, p38 MAPK and NF-κB [33]–[35], which upregulates gene expression of adhesion molecules and cytokines [21], [34], [36], [37]. We also found that less phosphorylation of p38 MAPKs and NF-κB, major downstream signaling factors of LOX-1, was observed in ischemic limb of LOX-1 KO mice than in that of WT mice (Fig. 4A a, b). In this experiment, inhibition of NF-κB activation by LOX-1 deletion caused less VCAM-1 (an adhesion molecule) expression in endothelial cells, which reduces macrophage infiltration into the ischemic tissue (Fig. 4A a, b, Fig. 5). Importantly, LOX-1 also plays a role as an adhesion molecule and then Infiltration of macrophages is supposed to be more significant in WT mice (upregulated expression of LOX-1, Fig. 3A) than in LOX-1 KO mice in the ischemic hindlimb [21]. It has been reported that LOX-1 activation also induced MCP-1 expression which is a NF-κB inducible cytokine and enhances infiltrated macrophage migration [34], [38], [39]. These findings suggest that LOX-1 activation and its upregulation in the ischemic hindlimb enhance the expressions of adhesion molecules on endothelial cells and the infiltration of macrophages into the ischemic tissues.

Interestingly, we also found that the expression of VEGF, the most powerful angiogenic factor, was significantly lower in the ischemic hindlimbs of LOX-1 KO mice in this experiment, but not the expression of VEGFR2 which is the key receptor of VEGF (Fig. 3B a, b). The precise mechanism of VEGF secretion via LOX-1 has not been elucidated yet, but it has been reported that oxidized phospholipids stimulate angiogenesis via autocrine mechanisms involving VEGF in advanced atherosclerotic lesions [40] and that chondrocytes stimulated by oxidized LDL via LOX-1 secrete VEGF [41]. As other groups reported that VEGF is secreted from infiltrated macrophages in ischemic tissues and contributes to neovascularizationan [2], [6], [27], [42], [43], we examined whether the expression of VEGF secreted from infiltrated macrophages decreased in the ischemic hindlimb of LOX-1 KO mice compared with WT mice. In our study, macrophage infiltration in ischemic tissue was impaired significantly in LOX-1 KO mice compared with that in WT mice. Dual immunofluorescence staining showed that infiltrated macrophages released VEGF protein and that the number of VEGF-positive macrophages was reduced in LOX-1 KO mice compared with that in WT mice, suggesting that deletion of LOX-1 provoked a decreased number of macrophages and VEGF production by macrophages, which disturbed angiogenesis and recovery of blood flow in the ischemic hindlimbs (Fig. 1, 2 and 5).

It has been also reported that HIF-1α, which expression was downregulated in the ischemic hindlimb of LOX-1 KO mice as well as Nox2, upregulates VEGF protein expression [3], [44]; therefore, it is likely that deletion of LOX-1 downregulates VEGF production via inactivation of HIF-1α and the suppressed VEGF production in infiltrated macrophages in LOX-1 KO mice decelerates angiogenesis after ischemia in this experiment.

In addition to p38 MAPK related to LOX-1 signaling, we also examined whether the deletion of LOX-1 has effects on other MAPKs, namely ERK and SAPK. Indeed, LOX-1 KO mice did not exhibit altered expression and phosphorylation of ERK and SAPK.

It has been reported that VEGF phosphorylates Akt and then phosphorylates eNOS [45]–[47]. Akt and eNOS have been subsequently reported to accelerate angiogenesis [46], [48], [49]. In the present study, both phosphorylated Akt and eNOS expression was suppressed in LOX-1 KO mice compared with that in the WT mice, as shown in Fig. 4B. Therefore, it is likely that reduced VEGF production caused less phosphorylation of Akt and eNOS and then suppressed angiogenesis and blood flow recovery.

There are study limitations to be considered. As we employed conventional knockout mice and did not use the bone marrow transplantation technique to evaluate the physiological functions of LOX-1 in ischemic limbs in our study, we can not clearly demonstrate which cell, such as macrophages, endothelial cells, smooth muscle cells and so on, is the most important for the decrease in angiogenesis via LOX-1.

Our results indicate that infiltrated macrophages producing VEGF (mainly ROS–HIF-1α–VEGF pathway) and upregulated expressions of adhesion molecules such as VCAM-1 (Nox2-ROS-NF-κB pathway) and upregulated LOX-1 itself on endothelial cells are important for angiogenesis in this experiment. Furthermore, we would like to establish a cell-specific knockout mouse model or system using bone marrow transplantation. This would give us more information to clarify the physiological functions of LOX-1.

## Conclusions

The findings of the present study are that deletion of LOX-1 1) decreased hindlimb blood flow after surgically induced ischemia; 2) decreased the number of microvessels: capillaries and arterioles; 3) down-regulated the expression of VEGF, Nox2, HIF-1α, VCAM-1 and generation of ROS; 4) decreased the phosphorylation of NF-κB (phosphorylation of p65 subunit) and p38 MAPK; 5) decreased the phosphorylation of Akt and eNOS; and 6) suppressed the migration of infiltrated macrophages that secreted VEGF protein.

These findings indicate that enhanced infiltration of macrophages producing VEGF and upregulated expressions of adhesion molecules such as VCAM-1 and LOX-1 itself on endothelial cells are important for angiogenesis in the ischemic hindlimb. Therefore, we concluded that LOX-1 plays an important role in promoting angiogenesis in the ischemic hindlimb.

## Materials and Methods

### Animals

LOX-1 KO mice with a C57/BL6J background were produced as described previously [22] and LOX-1 KO mice and control wild-type (WT) C57/BL6J strain mice were a kind gift from Dr. Tatsuya Sawamura (National Cerebral and Cardiovascular Center, Suita, Japan). Male LOX-1 KO mice and WT mice were used at the age of 12 weeks.

This study was carried out in strict accordance with the recommendations in the Guide for the Care and Use of Laboratory Animals of the National Institutes of Health (NIH Publication, 8th Edition, 2011). The protocol was approved by the Institutional Animal Research Committee of Gifu University (Permit Number: 17−68). All surgery was performed under sodium pentobarbital anesthesia. Mice were euthanized by cervical dislocation following sodium pentobarbital anesthesia (200 mg/kg, intraperitoneally, Sigma Chemical Co., St. Louis, MO, USA) until righting reflex was lost and their hindlimb tissues were excised and subjected to analyses such as morphological examination and Western blotting. All effort was made to minimize suffering.

### Surgical procedure of hindlimb ischemia

We prepared a hindlimb ischemia model in male WT mice and LOX-1 KO mice. Under sufficient anesthesia with sodium pentobarbital (50 mg/kg, intraperitoneally), local fur was removed with depilatory cream. A small incision was made on the right leg to expose the femoral vasculature and dual ligation of the femoral artery was performed at a point just below the inguinal ligament, as previously described [4].

### Laser Doppler blood flow analysis

After anesthesia, hair was removed from both legs using a depilatory cream, following which the mice were placed on a heating plate at 37°C for 10 minutes to minimize temperature validation. We measured the ischemic hindlimb (right)/nonischemic hindlimb (left) blood flow ratio by laser Doppler blood flowmetry (LDBF; Moor LDI, Moor Instruments, Wilmington, DE), which provides a non-invasive measurement of blood flow. Data acquisition was performed by a previously described method [4]. Briefly, the sedated mice were secured on a monochromatic surface and an area of 11×11 cm was scanned from the lower abdomen to the end of the toes. Color images were obtained and the ischemic hindlimb/nonischemic hindlimb blood flow ratio was determined. Perfusion of the hindlimb prior to surgery was compared to that at specified time points: postoperative days 3, 7, 14, 21 and 28.

### Determination of capillary density (CD) and arteriole density (AD)

Mice in each group were euthanized on postoperative day 28 by cervical dislocation following an overdose of sodium pentobarbital (200 mg/kg, intraperitoneally). The gastrocnemius muscles with ischemic area of the hindlimbs were harvested, and some of the central tissues were fixed in methanol and then embedded in paraffin. In order to detect CD and AD, sections 4 µm thick were incubated with the primary antibody to von CD31 (1∶100, abcam, Cambridge, MA) and α-Skeletal Muscle Actin (1∶100, α-SMA; abcam) for 2 h at room temperature, rinsed with PBS and incubated with biotinylated antimouse IgG antibody for 30 min. The slides were then incubated in avidin–biotin complex for 30 min followed by rinsing with PBS, then incubated in diaminobenzidine, and finally washed in distilled water and counterstained with hematoxylin.

Quantitative assessments of the number of CD31-positive capillaries (CD) or α-SMA-positive arterioles (AD) were made in 20 randomly chosen high-power fields (HPF, x200) from 5 sections using a multipurpose color image processor. CD and AD are also expressed as the number of CD31-or α-SMA-positive vessels per HPF.

### Evaluation of inflammatory cell infiltration

Mice were euthanized on postoperative day 7 by cervical dislocation following an overdose of sodium pentobarbital (200 mg/kg, intraperitoneally). The gastrocnemius muscles with ischemic area of the hindlimbs were harvested and embedded in OCT compounds. Cryostat sections of 4 µm thickness were mounted on silicone-coated slides. They were incubated overnight at 4°C with an anti-mouse VEGF mAb (Santa Cruz Biotechnology, Santa Cruz, CA) and with an anti-mouse macrophage mAb (F4/80; Santa Cruz Biotechnology) in a moist chamber. The slides were then incubated for 30 min at 37°C with an Alexa Fluor 488-conjugated anti-rat immunoglobulin M (IgM) antibody (Invitrogen Corporation, Camarillo, CA) to detect VEGF. Then, they were incubated further for 30 min at 37°C with Alexa Fluor 568-conjugated anti-mouse immunoglobulin M (IgM) antibody (Invitrogen Corporation) to detect macrophages. Slides were examined and photographed by fluorescence microscopy (BZ-8100; KEYENCE Corporation, Osaka, Japan). Infiltrated F4/80-positive macrophages and those co-stained with anti-VEGF mAb are expressed as the average number of immunopositive cells in 20 randomly chosen HPF (x200).

### Western blot analysis

Tissues (200 mg) from the center of the ischemic region of the hindlimbs were excised from three animals 7 days after ligation of femoral arteries and subjected to Western blot analyses. Proteins were separated and transferred to membranes using standard protocols, after which they were probed with antibodies against VEGF (Santa Cruz Biotechnology), Akt, endothelial nitric oxide synthase (eNOS), NF-κB p65 subunit, p38 mitogen-activated protein kinase (MAPK), extracellular signal-regulated kinase 1/2 (ERK), stress-activated protein kinases (SAPK), Actin (Cell Signaling, Danvers, MA), LOX-1, HIF-1α, VEGF, NADPH oxidase (Nox2), VCAM-1 (abcam). Activity of Akt, eNOS, NF-κB p65 subunit, p38 MAPK, ERK and SAPK was evaluated by Western blot for their phosphorylated forms using antibodies against the phospho-Akt, phospho-eNOS, phospho-NF-κB p65 subunit, phospho-p38 MAPK, phospho-ERK and phospho-SAPK (Cell Signaling). The blots were visualized using chemiluminescence (LumiGLO, Cell Signaling), and the signals of the bands were quantified with a Calibrated Imaging Densitometer (LAS-3000IR; FUJIFILM).

### Measurement of ROS generation

Tissue (100 mg) was washed with saline to remove as much blood as possible. We blotted the tissue with paper towels and then measured its weight. 500 µl sucrose buffer (0.25 M sucrose, 10 mM Tris, 1 mM EDTA, pH 7.4) was added and the sample was homogenized by using Teflon homogenizer and then centrifuged at 10,000 g for 60 min at 4°C, and the supernatant was transferred to a new tube. ROS generation was detected using the SOD Assay Kit-WST (Dojindo Molecular Technology, Japan) in a microplate reader according to manufacturer’s protocol.

### Statistical analysis

All data are expressed as means ± SD. Differences between groups were examined for statistical significance using 1-way ANOVA to compare all data. Statistical significance was accepted at a value of P<0.05.
